# Solvent Engineering for High-Performance PbS Quantum Dots Solar Cells

**DOI:** 10.3390/nano7080201

**Published:** 2017-07-28

**Authors:** Rongfang Wu, Yuehua Yang, Miaozi Li, Donghuan Qin, Yangdong Zhang, Lintao Hou

**Affiliations:** 1 Institute of Polymer Optoelectronic Materials & Devices, State Key Laboratory of Luminescent Materials & Devices, South China University of Technology, Guangzhou 510640, China; mswrf@mail.scut.edu.cn (R.W.); sendiege@163.com (Y.Y.); 2 Siyuan Laboratory, Guangzhou Key Laboratory of Vacuum Coating Technologies and New Energy Materials, Guangdong Provincial Key Laboratory of Optical Fiber Sensing and Communications, Department of Physics, Jinan University, Guangzhou 510632, China; zhangyangdong@stu2014.jnu.edu.cn; 3 School of Materials Science and Engineering, South China University of Technology, Guangzhou 510640, China; limz4994@sina.com

**Keywords:** colloidal quantum dots, solar cells, PbS, solvent engineering

## Abstract

PbS colloidal quantum dots (CQDs) solar cells have already demonstrated very impressive advances in recent years due to the development of many different techniques to tailor the interface morphology and compactness in PbS CQDs thin film. Here, n-hexane, n-octane, n-heptane, isooctane and toluene or their hybrids are for the first time introduced as solvent for comparison of the dispersion of PbS CQDs. PbS CQDs solar cells with the configuration of PbS/TiO_2_ heterojunction are then fabricated by using different CQDs solution under ambient conditions. The performances of the PbS CQDs solar cells are found to be tuned by changing solvent and its content in the PbS CQDs solution. The best device could show a power conversion efficiency (PCE) of 7.64% under AM 1.5 G illumination at 100 mW cm^−2^ in a n-octane/isooctane (95%/5% *v*/*v*) hybrid solvent scheme, which shows a ~15% improvement compared to the control devices. These results offer important insight into the solvent engineering of high-performance PbS CQDs solar cells.

## 1. Introduction

Colloidal quantum dots (CQDs) heterojunction solar cells that can be fabricated by simple solution-processing techniques are under intensive research due to their potential for low cost, air stable and large area efficient photovoltaic devices [1,2,3,4,5,6,7,8,9,10]. Among all kinds of CQDs, PbS CQDs allow bandgap tuning easily through the quantum size effect by changing the diameter of CQDs, which allows efficient light harvesting over a wide range of the visible/infrared wavelength in multiple junction solar cells formed by single material [11,12,13,14]. The most common and efficient PbS CQDs solar cells architecture consists of PbS CQDs active layer and TiO_2_ or ZnO electron acceptor layer to form depleted heterojunction. Comparing with the PbS CQDs solar cells in configuration of Schottky diode or normal device architecture, PbS/TiO_2_-depleted device with the inverted structure has many merits. For example, the placement of the junction on the illuminated side reduces the recombination rate of minority carriers [15]. On the other hand, the band alignment of PbS CQDs and TiO_2_ will block holes transfer to TiO_2_ at the interface. Thanks to the development of materials and device processing techniques, the power conversion efficiency (PCE) of CQD solar cells has increased from 0.00004% [16] in 2005 to certified 10.6% [17] in 2016. Due to the large surface to volume ratio in CQDs materials, substantial unsaturated dangling bonds will create electronic trap states, which will promote the recombination of charge carriers that is detrimental to the CQDs solar cells performance [13]. To overcome this drawback, a series of ligands strategies including organic, organic–inorganic hybrid or atomic ligands have been developed to passivate trap states of CQDs and a dramatic improvement in device performance is obtained [18,19,20,21]. Band alignment between the PbS CQDs and TiO_2_ is also critical for favoring electron injection to the conduction band of TiO_2_. For example, by doping TiO_2_ with Sb or Zr [22], great improvement of PCEs was obtained in CQD solar cells arising mainly from an increase in short-circuit current (*J*_sc_). Another way to improve the CQD solar cells performance is decreasing the recombination at the metal oxide-semiconductor interface. In this strategy, introducing passivation ligands [23,24,25,26] around CQD can increase the width of the depletion region and optimize the electron collecting efficiency. 

Improvements in PbS CQDs solar cells can further offer guidance on better understanding as well as eliminating the factors that affect device performance. As a high ratio of surface area to volume existed in PbS CQDs solar cells, reducing the number of recombination centers is a most important way for achieving high device performance [27,28,29,30,31,32]. In particular, the nature of the morphology and compactness of PbS CQDs thin film are key factors that determine solar cells performance. The evaporating rate, viscosity and dispersability of solvent will affect the aggregations and the compactness of CQDs film. In the previous research, however, few works have been focused on the solvent effects on the device performance. n-octane is widely used as a single solvent for the preparation of PbS CQDs solution. The boiling point of n-octane is as high as 125.7 °C, so postponed annealing at ~50 °C for up to 10 h must be carried out after the deposition of PbS CQDs film in order to remove the existed solvent. Most recently, Lan et al. [17] employed a co-solvent (toluene and dimethylformamide) to incorporate iodide on CQDs before device fabrication. They found that improved passivation was obtained, which resulted in a certified efficiency of 10.6%. Herein, for the sake of adjusting the evaporating rate and viscosity of n-octane, we selected n-hexane, n-heptane, isooctane, toluene, n-octane and their hybrid as solvent for the dispersion of PbS CQDs. As shown in Table 1, the boiling point for n-heptane, isooctane, toluene and n-hexane is 99, 98, 111 and 69 °C, lower than that of n-octane (125.7 °C). On the other hand, toluene has high polarity of 2.4 in comparison to other solvents used in this case. Devices with configuration of FTO (F doped SnO_2_)/ZnO/TiO_2_/PbS/Au are then fabricated by using these PbS CQDs solutions. We find that the performance of PbS CQDs solar cells is highly dependent on the content of solvent used. In single-solvent strategy, devices show high efficiency up to 6.0% in the case of toluene or n-octane solvent, while only 4~5% efficiency is obtained in the case of isooctane, hexane or heptanes solvent. It should be noted that, in the hybrid solvent scheme, the device performance can be further improved. For example, by adding less than 20% (*v*/*v*) isooctane into n-octane, the PCE is higher than that with pure n-octane and as high as 7.64% PCE is obtained in 5% isooctane/95% (*v*/*v*) n-octane mixture. Our research gives more insight into the solvent engineering for achieving high-performance PbS CQDs solar cells.

## 2. Experiment Procedure

### 2.1. Materials

Zinc acetate hydrate, ethanol amine, 2-methoxyethanol, titanium butoxide, triethanolamine, mercaptopropionic acid, hexamethyldisilathiane (TMS) and octyl-phosphine acid (OPA) were purchased from Aladdin (Shanghai, China). Oleic acid (OA), lead oxide, oleic amine (OLA), 1-octadece (ODE) were purchased from Alfa Aesar (Beijing, China). All chemicals were used directly without any further purification. 

### 2.2. Ti-Sols, Zn^2+^ Precursor and PbS CQDs Fabrication

The preparation of Ti-sols was carried out under ambient condition, as described in the literature [24]. Zn^2+^ precursor was prepared by adding zinc acetate and ethanolamine into the solution of 2-methoxyethanol with a concentration of 0.5 mol L^−1^. The resulting solution was refluxed at 60 °C for 2 h under ambient conditions. The final Zn^2+^ precursor was filtered through 0.45 μm filter before use. PbS CQDs were prepared by a typical synthetic procedure on a literature method [32]. A typical synthetic procedure was given below: in a three-necked flask, 0.45 g PbO, 1.5 mL OA, and 16.5 mL ODE were added. The mixtures were heated to 120 °C and pumped down for 6 h to remove any moisture and impurity. Then high purity (>99.99%) N_2_ was introduced to the flask; 0.20 mL TMS was mixed with 5 mL 1-octadece in a syringe and quickly injected into the mixture. The hotplate was removed away immediately after the injection and the mixture was cooled down to ~90 °C. At the same time, hybrid ligands were prepared by mixing 0.72 mmol CdCl_2_, 2 mL OLA, and 0.048 mmol OPA in a three-necked flask and degassed/refluxed at 90 °C. The hybrid ligands were injected into PbS CQDs mixture at this temperature and the reaction was cooled down to room temperature. After washing and centrifugation, PbS CQDs were dissolved into different solvents or hybrid solvent with a concentration of 25 mg/mL. 

### 2.3. Devices Fabrication

PbS/TiO_2_ CQDs heterojunction solar cells with a triple-layer structure, containing ZnO window layer, transparent TiO_2_ layer and PbS CQDs light absorber layer, were prepared according to a procedure reported before [32]. Firstly, FTO glass was cleaned in deionized (DI) water and isopropanol solution for 10 min under ultrasonic treatment. Zn^2+^ precursor was then deposited on cleaned FTO substrate at 2500 rpm for 15 s and put on a hot plate at 300 °C for 30 min to remove any organic solvent and impurity. One layer of Ti-sols was then spin-coated on the FTO/ZnO substrate at 2500 rpm for 15 s, following annealing at 500 °C for 1 h. After cleaning and drying, several drops of PbS CQDs in different solvents were put onto the FTO/ZnO substrate and spin-casted at 3000 rpm for 15 s. 3-Mercaptopropionic acid (MPA) was used for ligands exchange, as described in the literature [31]. It should be noted that the one-step coating PbS CQDs layer thickness for different solvents are different due to the difference in solvent volatilizing rate. In order to obtain PbS CQDs film with similar thickness of ~200 nm, for PbS/n-octane solution, four layers of PbS CQDs should be deposited, while five layers of PbS CQDs for PbS/toluene solution, five layers for PbS/isooctane solution and five layers of PbS CQDs for PbS/heptane solution. 

## 3. Results and Discussion

**Figure 1:**
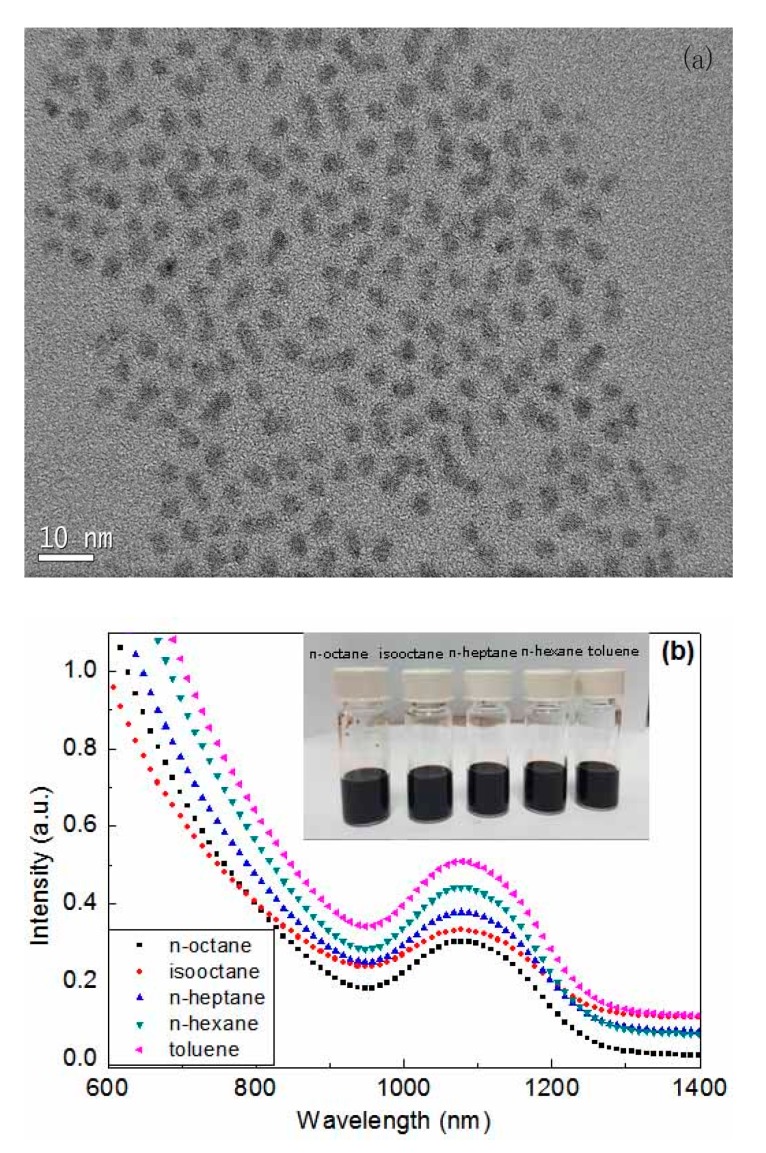
(**a**) TEM image of as prepared PbS CQDs (**b**) UV-absorption spectra of PbS CQDs in different solvents. CQD = colloidal quantum dots.

The TEM image (see Figure 1a) suggests that the CQDs are highly crystalline and homogeneous with average diameter 4.5 nm. The absorption spectra of PbS CQDs in different organic solvent are shown in Figure 1b. The PbS CQDs are purified by twice precipitating with acetone and ethanol. Following this, the products are re-dispersed in n-octane, isooctane, n-heptane, n-hexane and toluene with the same concentration of 25 mg/mL. It is mentioned that PbS CQDs can be well dispersed in all the above solvents even with a concentration up to 80 mg/mL, as shown in the inset of Figure 1b. The initial absorption spectrum peak for all PbS CQDs samples is located at ~1080 nm with full width at half maximum of ~150 nm, which indicates that these kinds of solvents do not have any changes on the physical properties of PbS CQDs. It is also found that there are almost no changes in absorption spectrum for the PbS CQDs samples after storage for several weeks, which implies that the PbS CQDs samples fabricated by the above means are very stable. This phenomenon can be explained by the successfully hybrid passivation strategy adopted in this case (PbS CQDs is passivated by CdCl_2_ + OPA + OLA), which is also confirmed by Sargent et al. [25]. 

**Figure 2:**
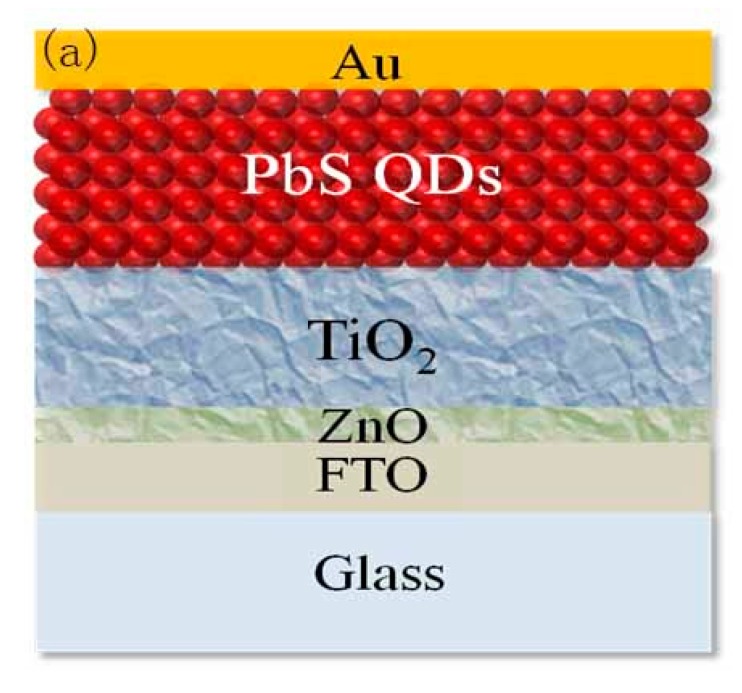

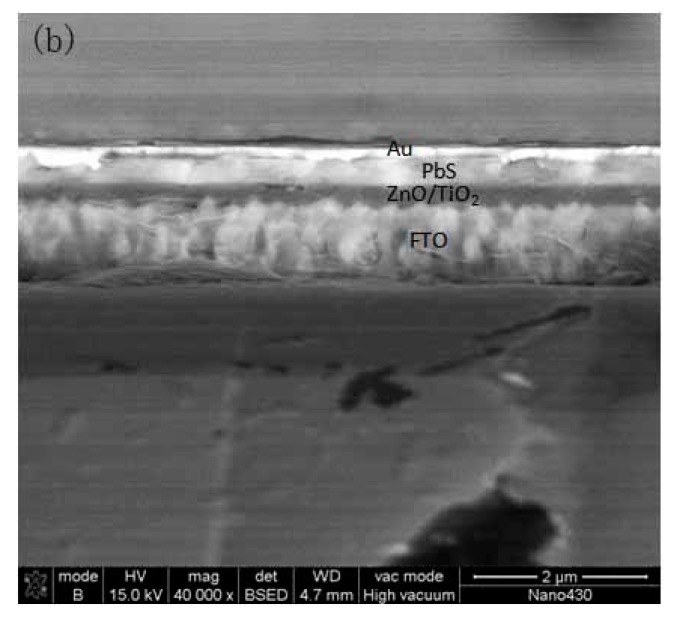
(**a**) Schematic of FTO/ZnO/TiO_2_/PbS/Au device architecture (**b**) The cross-sectional SEM images of a representative device.

The device configuration of PbS CQDs solar cells are provided in Figure 2a. A 40 nm layer of ZnO thin film is deposited on FTO substrate by thermal decomposition of zinc acetate to make a smooth and defects-free surface. Two layers of TiO_2_ film (~100 nm) is then deposited on ZnO to create an electron acceptor layer. PbS CQDs in different solvents are then spin-coated on top of the FTO/ZnO/TiO_2_ substrate. To eliminate the thickness effects on the solar cells performance, all PbS CQDs film are controlled to have similar thickness by using the same device processing technique. A typical cross-section SEM view of PbS CQDs solar cells device is shown in Figure 2b. The PbS CQDs active layer is very compact and homogeneous with the thickness around 200 nm.

**Figure 3:**
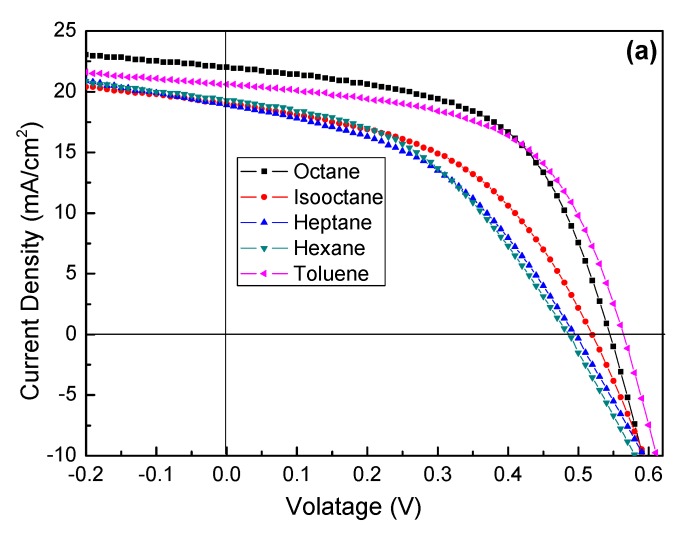

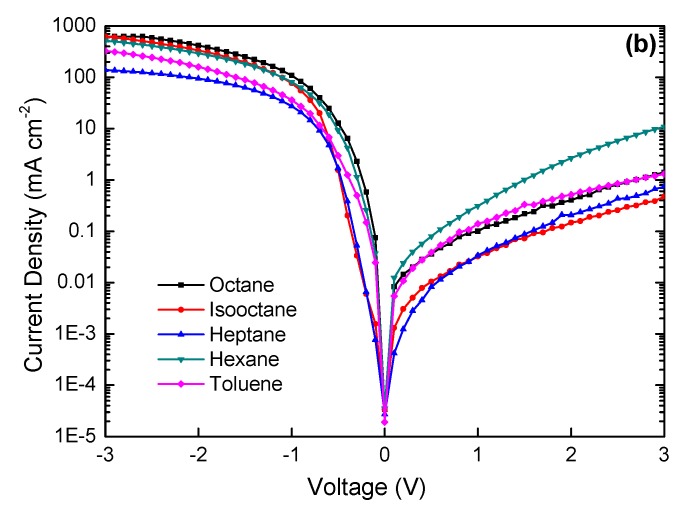
*J*–*V* characteristics of PbS/TiO_2_ solar cells fabricated by using different single solvent (**a**) under light (**b**) under dark.

The solvent has a great effect on thin film solar cells performance, which has been demonstrated in previous work [32,33,34,35,36,37,38]. A compact, defects-free and smooth surface is crucial for high-performance PbS CQDs solar cells. The viscosity and evaporating rate of solvent will affect the aggregation of PbS CQDs on the TiO_2_, so the device performance of PbS CQDs solar cells can be tailored by using different solvents and their hybrid. The current density–voltage (*J*–*V*) curves for PbS CQDs solar cells with single solvent measured under AM 1.5 G are presented in Figure 3a, while Figure 3b presents the dark *J–V* curves for these devices. The *J*_sc_, *V*_oc_, fill factor (FF), PCE value of devices determined from *J*–*V* curves are summarized in Table 2. The rectifier ratio calculated from dark *J–V* curve (Figure 3b) for n-octane, isooctane, n-heptane, n-hexane and toluene devices are 442, 1000, 188, 47 and 251, respectively. It is clear that the device fabricated using n-hexane solvent shows the lowest performance with *J_sc_* of 19.33 mA/cm^2^, *V_oc_* of 0.49 V, FF of 43.42 and PCE of 4.12%. On the other hand, n-heptane and isooctane devices show similar device performance with PCE ~5%. The toluene and n-octane devices show the highest performance. The *J_sc_*, *V_oc_*, FF and PCE for typical n-octane device are 23.50 mA/cm^2^, 0.56 V, 54.38 and 6.67%, respectively. It should be noted that the toluene device also shows high device performance with a PCE of 6.59% and a high *V_oc_* of 0.56 V. To the best of our knowledge, there are no reports on PbS CQDs solar cells fabricated by using solvent with relative high polarity such as toluene.

**Figure 4:**
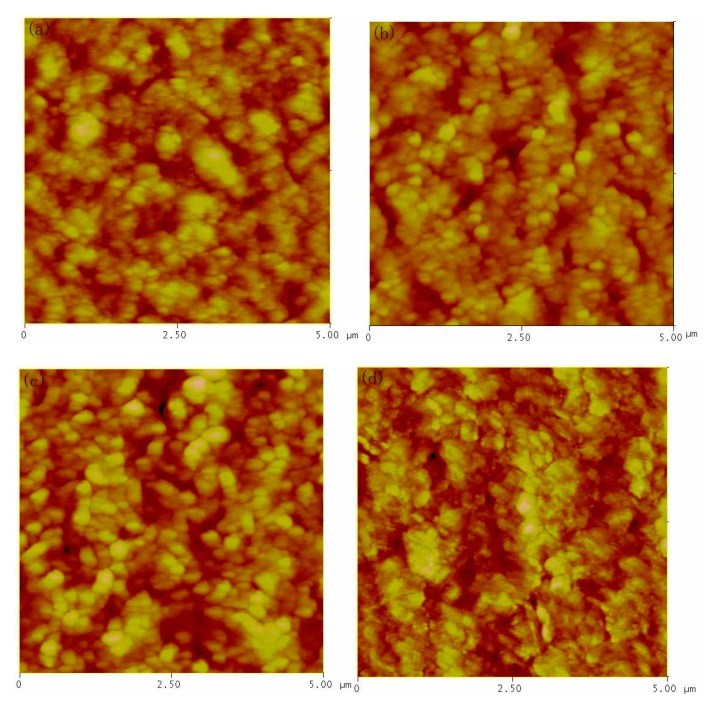
AFM (atomic force microscope) images of PbS CQDs thin films prepared by using different solvent (**a**) n-octane; (**b**) isooctane; (**c**) n-hexane; (**d**) toluene.

To achieve high device performance of PbS CQDs solar cells, a smooth, compact and homogeneous surface is necessary to decrease the carrier recombination in the interface and collect charges to the electrodes efficiently. To further investigate the interface properties of PbS CQDs active layer, we probe the surface morphology of PbS CQDs samples by atomic force microscope (AFM). As described in the experiment section, the PbS CQDs thin films are prepared by spin-coating PbS CQDs solution onto FTO/ZnO substrate. All samples AFM measurement consists of two layers of PbS CQDs. As shown in Figure 4, the surface morphology of all samples is uniform and smooth, but there are some differences in details. The particles size is small in the case of n-octane or isooctane sample while large aggregation is found in the case of n-hexane or toluene sample. The root mean square (RMS) value for n-octane, isooctane, n-hexane and toluene samples are 7.86, 14.20 nm, 19.57 nm and 11.70 nm, respectively (n-heptane samples has similar morphology as that of isooctane sample). It is clear that films fabricated from n-octane or toluene solvents show smooth and compact surface, which will reduce the number of grain boundaries and decrease leakage current. Thus, high PCE is obtained in the case of n-octane or toluene device because of low interface defect density, which is consistent to our *J*–*V* curves measurement. 

**Figure 5:**
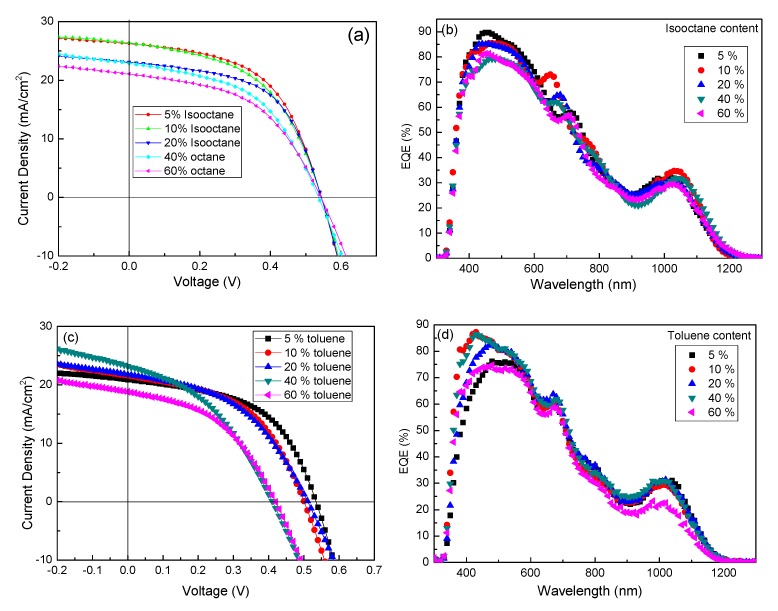
(**a**) *J–V* characteristic of PbS CQDs solar cells with n-octane/isooctane hybrid solvent and (**b**) the corresponding EQE spectrum; (**c**) *J–V* characteristic of PbS CQDs solar cells with n-octane/toluene hybrid solvent and (**d**) the corresponding EQE spectrum.

The solvent physical–chemical property has significant effect on solution-processed thin film solar cells, which had been reported in previous research. For further increasing the device performance, solvent mixtures are used to affect the evaporating rate, CQDs aggregation and finally the quality of CQDs thin film. We selected n-octane as the main solvent and isooctane or toluene as the doping solvent. As the two doping solvents have different boiling point and polarity, the addition of them in n-octane will surely change the evaporating rate and polarity of solvent mixture, etc. The polarity of n-octane will be increased with the addition of toluene. Figure 5a shows the *J–V* curves of PbS CQDs solar cells fabricated by using n-octane/isooctane mixture. From the data summarized in Table 3, one can see that the device performance is improved with the increase of isooctane content from 0 (*v*/*v*) to 20%, then drops down above 20%. It is noted that the champion device with PCE of 7.64% is obtained in the case of n-octane/isooctane (95%/5%, *v*/*v*) device, while this value is 6.67% for control device using pure n-octane, showing a ~15% increase mainly arising from a 15% increase in *J_sc_*. The PCE values of 10 and 20% isooctane doping n-octane devices are up to 7% respectively, which is higher than that of the control device. However, when isooctane exceeds 20%, device performance decreases almost linearly with the content increase of isooctane. The enhanced *J_sc_* for low content isooctane devices is confirmed by the external quantum efficiency (EQE) measurement shown in Figure 5b. Devices fabricated with low isooctane content show higher EQE in the wavelength region from 400 to 800 nm. This finding suggests that the carrier transfer efficiency is improved by adding a small amount of isooctane. On the contrary, descendant devices performance is found in the case of the hybrid solvent schemes based on n-octane and toluene. The *J–V* curves for n-octane/toluene hybrid solvent devices are shown in Figure 5c and the corresponding EQE spectrum is described in Figure 5d. It is clear that the device performance decreases linearly with the toluene content increase and all the hybrid solvent devices show lower PCE than that of n-octane or toluene single-solvent device, which is mainly arising from the decrease in *V_oc_* and FF of devices. We speculate that low-quality CQDs thin film may be obtained in this case due to large difference in polarity for n-octane/toluene hybrid solvent.

**Figure 6:**
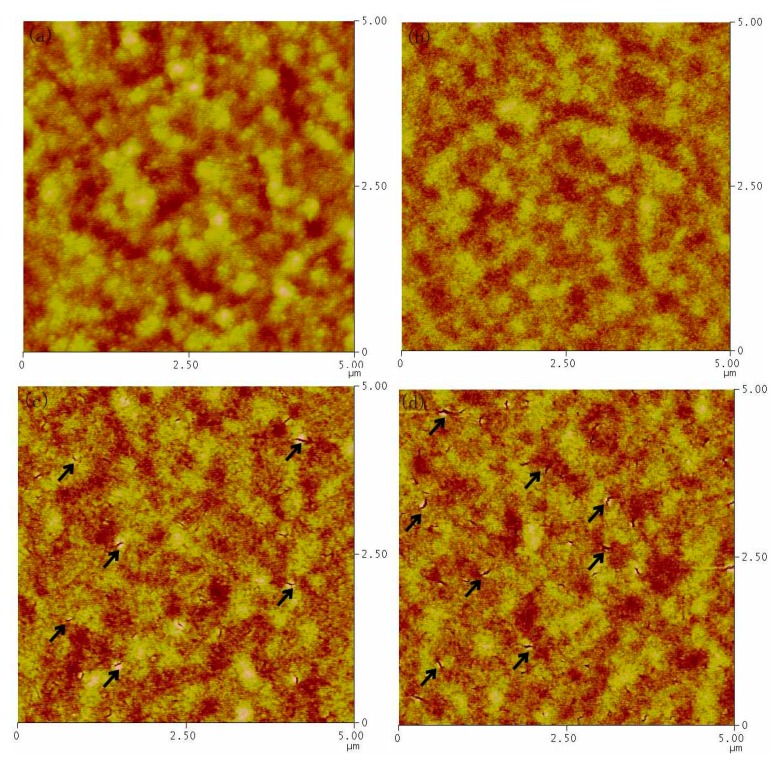
AFM (atomic force microscope) images for PbS CQDs thin film fabricated by using different n-octane/isooctane solvent (**a**) 5% isooctane (**b**) 10% isooctane (**c**) 20% isooctane (**d**) 40% isooctane.

**Figure 7:**
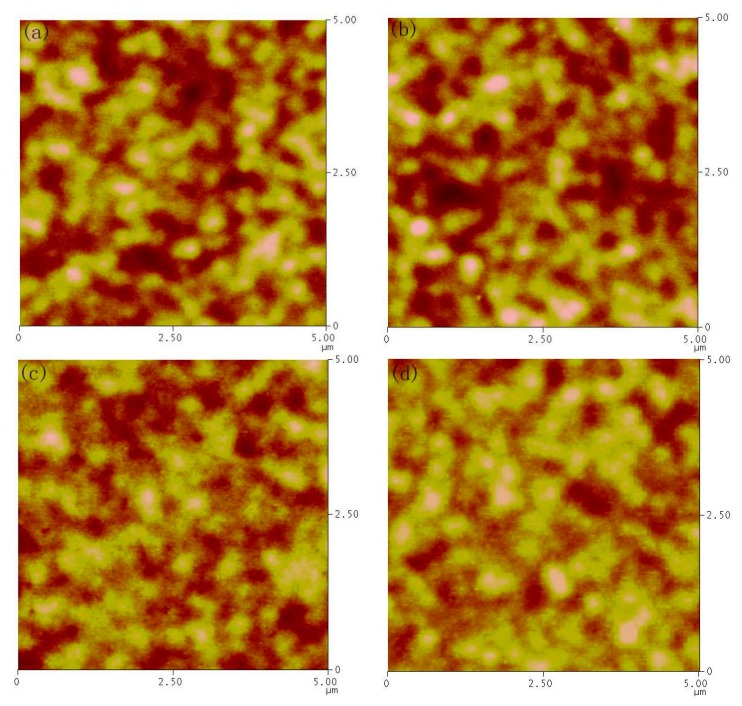
AFM (atomic force microscope) images for PbS CQDs thin film fabricated by using different n-octane/toluene solvent (**a**) 5% toluene (**b**) 10% toluene (**c**) 20% toluene (**d**) 40% toluene.

To investigate the morphology changes with different solvent contents, AFM measurement is carried out for PbS CQDs thin film prepared by using n-octane/isooctane and n-octane/toluene mixture. As shown in Figure 6, the thin film is homogeneous and compact for all samples with root-mean-square (RMS) value ~7.5 nm. However, the domain size is quite different in detail for thin films fabricated with different n-octane/isooctane content. In the case of low isooctane content, large aggregation of PbS CQDs is found with isooctane content increase from 5 to 10% (Figure 6a,b). On the contrary, when the isooctane content is up to 20% (60% samples have similar morphology as that of 40% samples), a lot of cracks appear in the whole thin film (see the arrow pointing). The cracks become bigger with the isooctane content increase from 20 to 40%. As Au will be deposited on the PbS CQDs thin film, large cracks may result in more interface defects, which will increase interface carrier recombination and low *J*_sc_ is expected in this case. However, it should be pointed out that the width of cracks is around 10 nm, much lower than the thickness of the CQDs active layer (around 200 nm), so it does not result in short, but a decreased in device performance. Furthermore, based on the above discussion, the increase in device performance for low isooctane content mixture is a result of compact and large aggregation derived from fast solvent mixture evaporation rate, while excessive isooctane content can result in cracks and lower device performance. It is interesting that the morphology of n-octane/toluene samples is quite different from those of n-octane/isooctane samples. As shown in Figure 7, no cracks are found in all CQDs thin film with different toluene content. The morphology is not homogeneous with RMS around 13.5 nm and some holes are found in all samples. The large fluctuation may result in large interface defect, which will lead to low device performance.

## 4. Conclusions

In conclusion, different solvents or their hybrid are developed to fabricate PbS CQD solar cells. The device performance can be tailored by selecting different solvents or changing the solvent mixture content. In the case of single-solvent strategy, high PCE up to 6.0% is obtained in the case of n-octane or toluene devices, while PCE below 5% is obtained for isooctane, heptane or n-hexanal devices. By using hybrid solvent, the device performance can be further tailored. PCE as high as 7.64% is obtained in the case of 5% isooctane/n-octane device, which is ~15% higher than the control devices. The changes in device performance mainly originated from different solvent physical-chemical properties, which affect the morphology of final PbS CQDs thin film. We believe that if an optimized PbS CQDs film, an optimized device structure or optimized device processing technics is presented, the device performance can be further increased. Our results present an effective way to improve the performance of solution-processed PbS CQDs solar cells. 

## Figures and Tables

**Table 1:** Summarized properties of different solvent.

Solvent Name	Polarity	Viscosity (Pa·s)	Boiling Point (°C)	Absorption Wavelength (nm)
n-octane	0.06	0.53	125	200
isooctane	0.10	0.53	99	210
heptane	0.20	0.41	98	200
n-hexane	0.06	0.33	69	210
toluene	2.40	0.59	111	285

**Table 2:** Photovoltaic parameters obtained from the *J–V* curves (Figure 3a) for solar cells with different single solvent.

Solvent	Power Conversion Efficiency (PCE) (%)	*J_sc_* (mA cm^−2^)	*V_oc_* (V)	Fill Factor (FF) (%)
n-octane	6.67	22.04	0.54	56.04
isooctane	4.62	19.00	0.52	46.76
heptane	4.69	21.63	0.52	41.70
n-hexane	4.12	19.30	0.49	43.57
toluene	6.59	20.60	0.56	57.13

**Table 3:** Photovoltaic parameters obtained from the *J*–*V* curves (Figure 5a,c) for solar cells with n-octane/isooctane and n-octane/toluene hybrid solvent.

**Isooctane/n-octane (*v*/*v*)**	**PCE (%)**	***J*_sc_ (mA cm^−2^)**	***V_oc_* (V)**	**FF (%)**	**R_s_ (Ω × cm^−2^)**	**R_sh_ (Ω × cm^−2^)**
5%	7.64	26.29	0.54	53.82	4.97	205.55
10%	7.26	26.38	0.54	50.93	5.47	109.21
20%	6.99	23.10	0.54	56.04	4.83	173.90
40%	6.08	22.90	0.54	49.17	7.02	104.33
60%	5.64	21.10	0.53	50.43	7.94	132.37
**Toluene/n-octane (*v*/*v*)**	**PCE (%)**	***J*_sc_ (mA cm^−2^)**	***V_oc_* (V)**	**FF (%)**	**R_s_ (Ω** **× cm^−2^)**	**R_sh_ (Ω** **× cm^−2^)**
5%	5.84	20.87	0.53	52.80	9.29	131.23
10%	5.31	21.57	0.50	49.24	11.37	109.94
20%	5.09	21.74	0.51	45.91	12.19	104.65
40%	3.85	23.20	0.41	40.48	8.20	55.56
60%	3.51	18.80	0.42	44.45	8.52	100.00
